# Wearable Fabric Loop Sensor Based on Magnetic-Field-Induced Conductivity for Simultaneous Detection of Cardiac Activity and Respiration Signals

**DOI:** 10.3390/s22249884

**Published:** 2022-12-15

**Authors:** Hyun-Seung Cho, Jin-Hee Yang, Sang-Yeob Lee, Jeong-Whan Lee, Joo-Hyeon Lee

**Affiliations:** 1 Institute of Symbiotic Life-TECH, Yonsei University, Seoul 03722, Republic of Korea; 2 Department of ICT Convergence, Engineering, Konkuk University, Seoul 27478, Republic of Korea; 3 Department of Clothing & Textiles, Yonsei University, Seoul 03722, Republic of Korea

**Keywords:** fabric loop sensor, sensor configuration, cardiac activity signal, respiration signal, clothing structure, apparel form-wearable platform

## Abstract

In this study, a noncontact fabric loop sensor based on magnetic-field-induced conductivity, which can simultaneously detect cardiac activity and respiration signals, was developed and the effects of the sensor’s shape and measurement position on the sensing performance were analyzed. Fifteen male subjects in their twenties wore sleeveless shirts equipped with various types of fabric loop sensors (spiky, extrusion, and spiral), and the cardiac activity and respiratory signals were measured twice at positions P2, P4, and P6. The measurements were verified by comparing them against the reference electrocardiogram (ECG) and respiratory signals measured using BIOPAC^®^ (MP150, ECG100B, RSP100C). The waveforms of the raw signal measured by the fabric loop sensor were filtered with a bandpass filter (1–20 Hz) and qualitatively compared with the ECG signal obtained from the Ag/AgCI electrode. Notwithstanding a slight difference in performance, the three fabric sensors could simultaneously detect cardiac activity and respiration signals at all measurement positions. In addition, it was verified through statistical analysis that the highest-quality signal was obtained at the measurement position of P4 or P6 using the spiral loop sensor.

## 1. Introduction

Recently, proactive prevention and healthcare have emerged in response to the increasing burden of medical expenses. This is due to an aging population and an increase in chronic diseases, as well as the rising interest in leading a healthy life. As income levels rise and interest in improving health and quality of life grows, the healthcare paradigm is shifting from a treatment-centered approach to one based on the principles of 4P (prevention, prediction, personalization, participation). Accordingly, personal health management (medical) devices to measure health biometric information and wearable technology to measure and monitor biosignals have been developed. A major trend in technology for collecting and monitoring personal health information is biosignal monitoring, representing the convergence of smart textiles, clothing design, and biosignal acquisition technology. An electrocardiogram (ECG) signal carries biometric information that reflects the state of heart activity; it has a regular cycle and is generated with a regular rhythm. However, a change in the state of activity or the occurrence of a lesion changes the characteristics of the period and waveform. Therefore, by analyzing the components of the ECG signal, it is possible to estimate the presence or absence of abnormalities in the heart or the site of the lesion. Similarly, it is possible to detect abnormalities in the pathological and physiological mechanisms of the heart and use them to diagnose heart disease. Therefore, the possibility of detecting biosignals, such as heart activity and respiration signals anytime and anywhere in daily life in an unconstrained and unconscious way can provide very important personal health data.

However, it is impractical to have 12 clinical-standard induction electrodes attached to the entire body for 24 h to detect ECG signals in daily life. Therefore, efforts have been made to develop wearable platforms equipped with two or three electrodes for acquiring measurements using an applied induction method, which can detect a part of the ECG rather than the entire waveform as a representative cardiac activity signal.

Most bands and clothing-attached electrodes for measuring cardiac activity and respiration developed in previous studies utilize contact-sensing methods. These can measure cardiac activity only in a state where certain parts of the body and the electrodes are completely compressed and in contact [1,2,3,4,5,6,7,8,9,10,11,12,13,14,15], which makes it difficult to acquire unconstrained and unconscious biosignals. To overcome this problem, some studies have been conducted on cardiac activity measurements using the noncontact capacitive method and magnetic-field-induction conductivity [16,17,18,19,20,21,22,23].

Koo et al. [22] analyzed the effect of the position of a textile-based inductive coil sensor on the measurement of the heart rate using magnetic-field-induced conductivity sensing methods. They reported that the cardiac activity signal measured using a noncontact inductive capacitive sensor was affected by respiration. Furthermore, the signal that envelops this cardiac activity signal shows very high agreement with the respiratory rate signal measured using a clinical device. Based on this, we attempted to simultaneously measure the cardiac activity and respiration signals using a noncontact inductive capacitive sensor. The changes in the heart volume in the chest tissue and position of the heart according to the exhalation and inspiration of the lungs were detected using eddy currents induced from surrounding objects by magnetic fields [24]. A method was devised to measure the cardiac activity and respiration signals simultaneously as a system. To this end, a noncontact sensor based on magnetic-field-induced conductivity was implemented in three loop shapes using fabric. Subsequently, the cardiac activity and respiration signal detection performance were analyzed according to the measurement position. In addition, we attempted to identify the most suitable sensor shape and measurement position to realize a wearable device that can acquire measurements of cardiac activity and respiration signals anytime and anywhere without being disturbed by movement.

## 2. Wearable Technology for Cardiac Activity and Respiration Signal Measurements

### 2.1. Cardiac Activity Signal

The most typical type of textile electrode for wearable cardiac activity sensing is the contact-type textile electrode. It acquires cardiac activity signals only when the skin and electrodes are in contact. These electrodes have the limitation of the signal acquisition being hindered or measurement noise being observed if there is a gap between the skin and electrode or the position of the contact point changes owing to the movement of the wearer. Cardiac activity signals measured using contact-type textile electrodes are typically based on the resistive or piezoelectric measurement principle. Song et al. [3] developed contact-type textile electrodes fabricated in a jacquard weaving style using silver-based metallic yarn for measuring heart-activity signals. Silver-covered yarn was used on the weft of the jacquard-woven electrodes to weave a double cloth. Subsequently, they implemented electrodes with and without conductive fillers inserted in the space between the layers of the double cloth. Furthermore, they employed two types of electrodes, one with a conductive paste coated on its back and one without. They fabricated four types of electrodes based on various combinations of electrode configurations (convex and flat type) and the application of conductive paste (applied and not applied) and compared the heart-activity-signal sensing performance of successive electrodes. The sensing performance was best when the heart-activity signals were measured using a convex-type electrode coated with conductive paste, which yielded the highest and most statistically significant signal-to-noise ratio (SNR) compared with the other three types of electrodes. Cho et al. [4] developed silver yarn-embroidered textile electrodes to evaluate the ECG sensing performance of each garment type and designed four types of biometric-signal-sensing smart garments. Through ECG measurements, they assessed the displacement of the electrodes in the stationary and moving states and analyzed the SNRs of the ECG signals. The analysis showed that among the four types of garments, the “cross-type” garment was the most effective for ECG measurements. Cho et al. [14] analyzed the effect of the structure of a contact-type textile electrode for detecting cardiac activity signals on the sensing performance. To measure the heart-activity signal, six types of contact-type textile electrodes, where the size and configuration of the electrodes were manipulated using a computer embroidery method, were attached to the chest band to detect the heart-activity signal using the modified Lead II method. In terms of the configuration of the contact-type textile electrode, the signal quality of the three-dimensional electrode was better than that of the flat electrode. However, no significant performance differences were found in the acquisition of cardiac activity signals according to the size of the three electrodes.

Studies on noncontact-type textile electrodes for heart-activity signal sensing include a study on a noncontact pulse measurement system of the radial artery using loose contact-type heart-rate-sensing textile electrodes and Colpitts oscillator capacitance [20,25]. This sensing method has the limitation of being difficult to measure the signals while the experimental subject is moving because it is sensitive not only to heart-activity signals but also to noise; in the case of a person with a thick fat layer in the wrist, the detected signals are very weak, making it difficult to distinguish between meaningful signals and noise. Another measurement principle of noncontact-type textile electrodes for heart-activity signal sensing is magnetic-field-induced conductivity. This method is relatively less affected by motion than electric-field-based heart-rate sensing methods. The underlying measurement principle is that a time-varying magnetic field applied to stimulate tissues in the body (e.g., muscles) generates eddy currents, and the magnetic field induced by the generated currents triggers a change in the inductance of the excitation coil, which appears as a change in the oscillation frequency [26].

Koo et al. [22] analyzed the effect of a textile-based inductive coil sensor on heart-rate measurements. Through a pilot test, they explored eight potential positions for the inductive coil sensor and selected three sensor positions. The heart-activity signals at these three positions were measured simultaneously, along with the reference signals (Lead II ECG signals). A comparison of the R-peak position between the reference signal and textile-based inductive coil sensor signals obtained at the three positions showed that the quality of the heart-activity signals obtained at “P3”, a sensor located 3 cm away from the center front line on the chest circumference line, was the highest. These results indicate that the position of the textile-based inductive coil sensor significantly affects the quality of the measurement results and the “P3” position is the most suitable for the measurement of heart-activity signals based on the magnetic-field-induced conductivity sensing principle.

### 2.2. Respiration Signal

The respiratory rate can be measured by converting these physical changes into electrical signals based on the changes in volume of the chest and abdomen that occur during respiration [27]. Respiratory measurement methods developed in wearable forms involve inductance, strain-gauge, capacitive, and piezoelectric plethysmography. Inductance plethysmography is a respiration measurement method that uses sensors on the chest and abdomen to measure the change in inductance according to the volume change that occurs during respiration [28,29,30].

Lee et al. [13] proposed a sensing method for a three-dimensional respiration-rate sensor based on the change in the surface area of the sensor. They evaluated the performance of a fabric-based three-dimensional respiration-rate sensor and a design compatible with clothing. Yang et al. [15] fabricated three types of respiration-rate sensor belts (2D, 3D soft-structure, and 3D hard-structure belts) consisting of a sensor and sensor support. The sensing performance was evaluated for each of the three respiration-rate sensors. The results of the experiment showed that the 3D wearable sensor based on the variable opening/closing structure was the most suitable for respiration-rate sensing. Based on this, a three-dimensional respiratory rate sensor and clothing structure to support the variable opening/closing-type sensor and minimize the baseline drift of the sensor were devised. Cho et al. [14] studied the effects of the type of fabric-based strain-gauge sensor and measurement location on the respiratory rate detection performance. They developed a chest belt-type wearable platform that could monitor various biosignals in real time without interfering with movement during daily activities. The results of the experiment showed that the respiratory rate was most effectively detected when the measurement was conducted with a carbon-nanotube-coated strain-gauge sensor in the chest or abdomen. Yang [11] fabricated an inductance fabric sensor by embroidering silver threads on fabric and verified the feasibility of measuring cardiac activity and respiration signals. In addition, the optimal measurement position was derived and a garment equipped with an inductance fabric sensor was designed. Kim [10] developed a strain-gauge-type fabric respiration sensor coated with transparent conductive oxide and multi-call carbon nanotubes and examined the measurement performance. By analyzing human motion through motion capture and measuring the signal sensitivity for each position of the sensor, the appropriate measurement location was selected, and a suitable clothing design was proposed. Min et al. [31] developed a capacitive-fiber pressure sensor and explored its measurement capability. A significant correlation coefficient was observed in the measurements acquired using the wearable capacitive-fiber pressure sensor and a flow meter. Cho and Min [32] evaluated the signal accuracy and wearability according to the measurement position of the capacitive-fiber pressure sensor. They provided recommendations for the selection of a suitable measurement position for the sensor and the design of apparel carrying the sensor. Son et al. [33] proposed the structure of a piezoelectric respiration rate sensor fabricated using a polyvinylidene fluoride film, which is a piezoelectric polymer material and explored the possibility of measuring the piezoelectric respiration-rate sensor using a respiration signal generation simulator.

## 3. Principle of Heart-Rate and Respiration Rhythm Measurements Using Induced Eddy Current on the Thorax

When an alternating current is applied to the fabric loop sensor, a primary time-varying field is formed around the sensor. When the fabric loop sensor is adjacent to biological tissue, an electric field is generated according to Faraday’s law of electromagnetic induction, as shown in Equation (1). B is the magnetic field density of the primary time-varying field generated by the fabric loop sensor, ∇× is the Curl operator, and E is the electric field generated by Faraday’s electromagnetic induction law.

(1) $$\nabla \times E=-\frac{\partial B}{\partial t}$$

**Figure 1:**
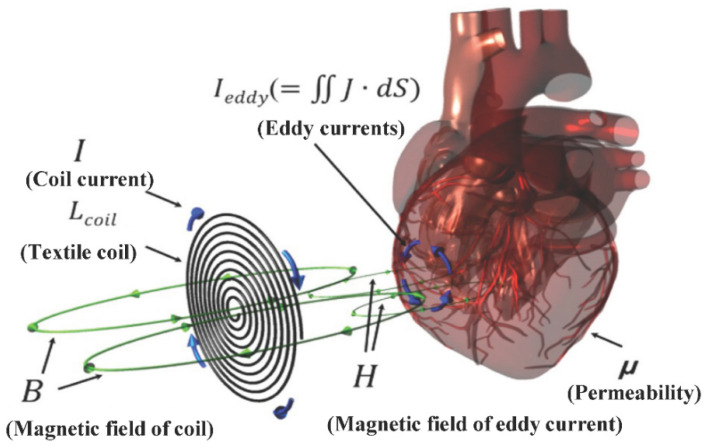
Current and magnetic field induced by the external magnetic field.

Accordingly, as shown in Figure 1, the eddy current ($I_{\mathrm{eddy}}$) is generated in the living tissue and a secondary induced magnetic field is formed according to Equation (2) in a direction opposite to the primary time-varying field generated by the sensor. H represents the secondary induction field generated in the biological tissue and J represents the current density generated by the sensor. ε is the permittivity of living tissue.

(2) $$\nabla \times H=J+\frac{\partial }{\partial t}\varepsilon E$$

As a result, the secondary induced magnetic field generated by $I_{\mathrm{eddy}}$ affects the primary time-varying field generated by the coil, which is shown by the influence on the induction of the fabric loop sensor. When the biological tissue changes, such as during cardiac activity or respiration, $I_{\mathrm{eddy}}$ generated by the biological tissue and H change, as well as the effect on the induction capacity of the fabric loop sensor. The effect of the magnetic field on the human body decreases significantly as it enters the body. The transmission depth of the magnetic field is set to a position reduced to 37% of the magnetic field’s effect on the skin and its range can be measured through sensors. The general formula is shown in Equation (3). δ, f, μ, and σ represent the transmission depth, frequency, permeability, and conductivity, respectively [34].

(3) $$\delta =\frac{1}{\sqrt{\pi f\mu \sigma }}$$

**Figure 2:**
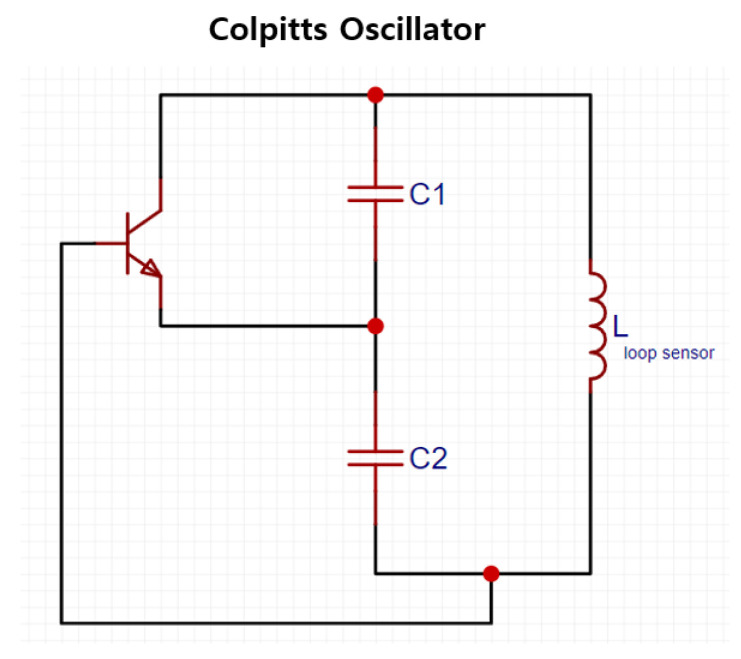
Colpitts oscillator circuit.

Figure 2 shows a circuit that generates a sinusoidal signal with a frequency ($f_{c}$) that is calculated using Equation (4) through the LC tank with a Colpitts oscillator. When a fabric loop sensor is used as an inductor ($L_{loopsensor}$) of the Colpitts oscillator, the change in the sensor’s induction capacity caused by cardiac activity or respiration appears as a frequency change in the sinusoidal signal generated by the oscillator.

(4) $$f_{c}=\frac{1}{2\pi \sqrt{\frac{L_{loopsensor}C_{1}C_{2}}{C_{1}+C_{2}}}}$$

**Figure 3:**
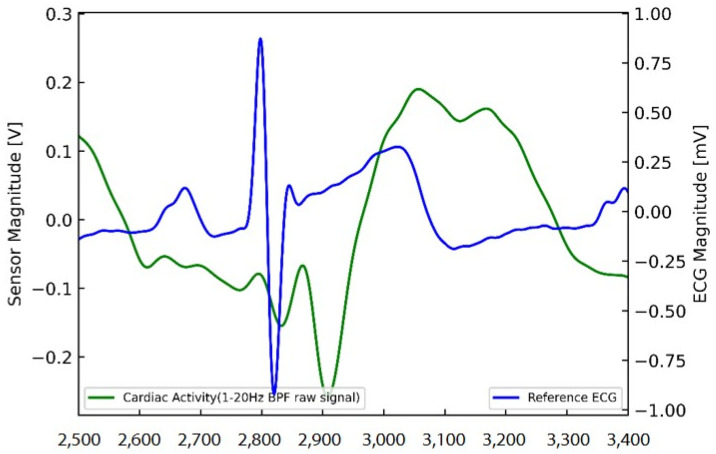
Time delay between ECG and fabric loop sensor signals.

The heart performs mechanical activities, such as contraction and relaxation of the myocardium generated by its electrical activity, and circulates blood throughout the body. In general, an electrical signal is generated in the sinusoidal node (SA) of the heart, which first generates atrial contraction, and then conducted to the atrioventricular node (AV). Electrical signals conducted by AVs pass through the HIS bundle and branches to the Purkinje fiber, causing ventricular contraction. An electrocardiogram (ECG) is a measure of the electrical activity of the heart. However, in this study, the measurement method is based on signals from mechanical activities due to contraction and relaxation and not from the electrical activity that occurs in the heart. As the electrical signal is generated first and the mechanical activity follows, there is a delay between the measured and the ECG signals, as illustrated in Figure 3.

## 4. Materials and Methods

### 4.1. Implementation of Fabric Loop Sensors

Three types of fabric loop sensors (spiky loop sensor, extrusion loop sensor, and spiral loop sensor) were prepared using computer machine embroidery with 30 strands (900 Ω/m) of 40 denier silver yarn. A fabric loop sensor was fabricated in a spiral shape, whose cardiac activity sensing performance has been validated in previous studies [19,20,21,22,23]. In addition, fabric loop sensors were fabricated with two other shapes—extrusion loop and spikey loop—by modifying the spiral loop sensors. The shapes and specifications of the three types of fabric loop sensors are listed in Table 1. To ensure a stable shape of the fabric sensor, it was post-processed using the heat-and-press method and the signal line was connected with a snap button (Table 1).

### 4.2. Hardware

**Figure 4:**
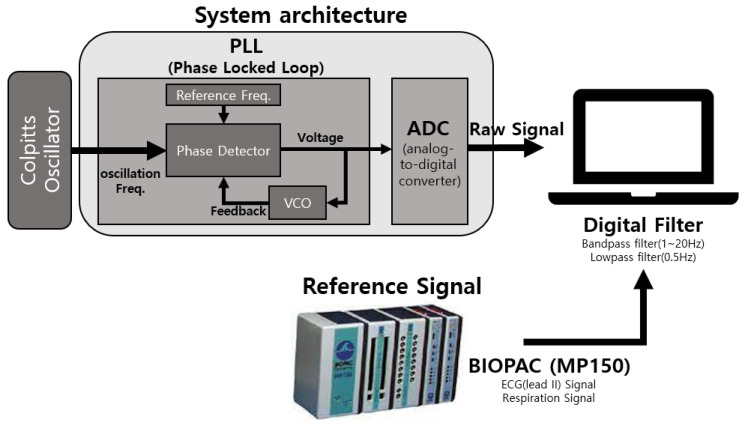
Hardware system structure.

Figure 4 shows the overall hardware system structure. The hardware configuration was largely composed of a Colpitts oscillator that generates a sinusoidal signal through a coil and a phase-locked loop (PLL), which converts the frequency change in a signal generated through the Colpitts oscillator into a signal. The supply voltage was 5V (Vcc). The Colpitts oscillator generates a sinusoidal signal ($f_{c}=2.5–3\;\mathrm{MHz}$) based on the LC configured in a structure of the LC tank using a fabric loop sensor as L. In this study, C was fixed and the fabric loop sensor was used as L to make the change in inductance of the fabric loop sensor appear as a change in the frequency of the oscillating signal. The PLL unit compares the input and reference frequencies with a phase detector to transmit the frequency difference as a voltage, converts the output voltage into a frequency using a voltage-controlled oscillator (VCO), and adjusts the output frequency through feedback. In this study, the frequency of the input signal from the Colpitts oscillator was compared with the reference frequency set by the phase comparator ($f_{ref}=2.5–3\;\mathrm{MHz}$), and it output a voltage equivalent to the frequency difference. By converting this change in voltage using an ADC, the raw signals (PLL) of heart activity and respiration can be detected.

### 4.3. Measurement Method

**Figure 5:**
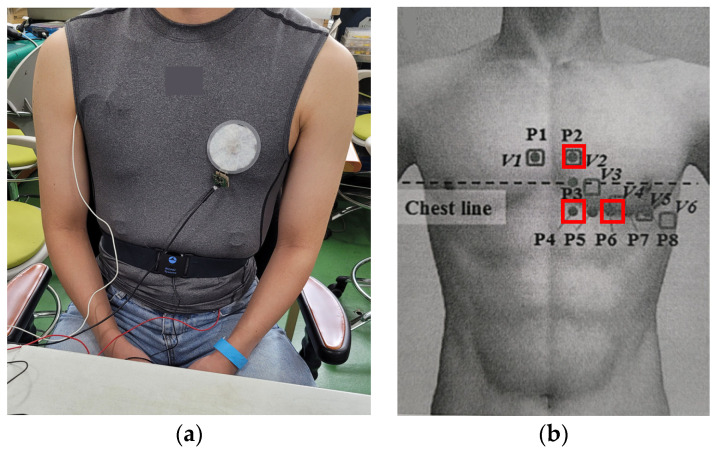
Cardiac activity and respiration signal acquisition experiment: (**a**) experimental image, (**b**) measurement position.

This experiment was conducted on 15 healthy male subjects in their twenties in a laboratory environment. First, each subject was allowed to rest in a sitting state and cardiac activity signals were obtained from the P2, P4, and P6 sites using the three types of fabric loop sensors. One electrode for each of the developed sensors was sequentially attached to a sleeveless sports shirt, which was then worn by the subject. The experiment was designed to repeatedly measure the cardiac activity signals (measured twice for 20 s each) at three sites—P2, P4, and P6—using the developed sensors. Because the signal measurement was based on a coil’s magnetic field, the experiment was conducted so that the conditions that could affect the test environment were limited. In addition, ECG and respiration signals were measured using BIOPAC^®^ (MP150, ECG100B, RSP100C) as reference signals to verify the reliability of the signals measured using the developed sensors. The ECG was conducted by attaching a clinical Ag/AgCl electrode to the human body using the standard Lead II method, and the respiratory signal was measured using a breathing belt worn on the lower abdomen (Figure 5).

### 4.4. Data Collection and Analysis

Cardiac activity and respiration signals were simultaneously measured in 15 subjects by repeating the experiment twice for each electrode type and location. The signals detected by the three fabric loop sensors were sampled at 1 kHz using BIOPAC^®^ (MP150, ECG100B, RSP100C). In addition, to quantitatively compare the cardiac activity detection performance according to the shape of each fabric loop sensor, the SNR (Signal-to-Noise Ratio) of the detected signal was calculated using Equation (5). This is the ratio of the signal power of the 1 to 20 Hz band, where the heart signal is distributed, to the signal power of the remaining noise band. Frequency analysis was performed using fast Fourier transform. By normalizing the detection results of the cardiac signal obtained by the fabric loop sensor, the differences caused by the intrinsic characteristics of each subject were reduced.

(5) $$\mathrm{SNR}=\frac{\sum_{f=1\mathrm{Hz}}^{20\mathrm{Hz}}P_{signal}}{\sum_{f=20\mathrm{Hz}}^{500\;\mathrm{Hz}}P_{noise}}$$

## 5. Results and Discussion

### 5.1. Evaluation of Sensing Performance through Qualitative Analysis

**Figure 6:**
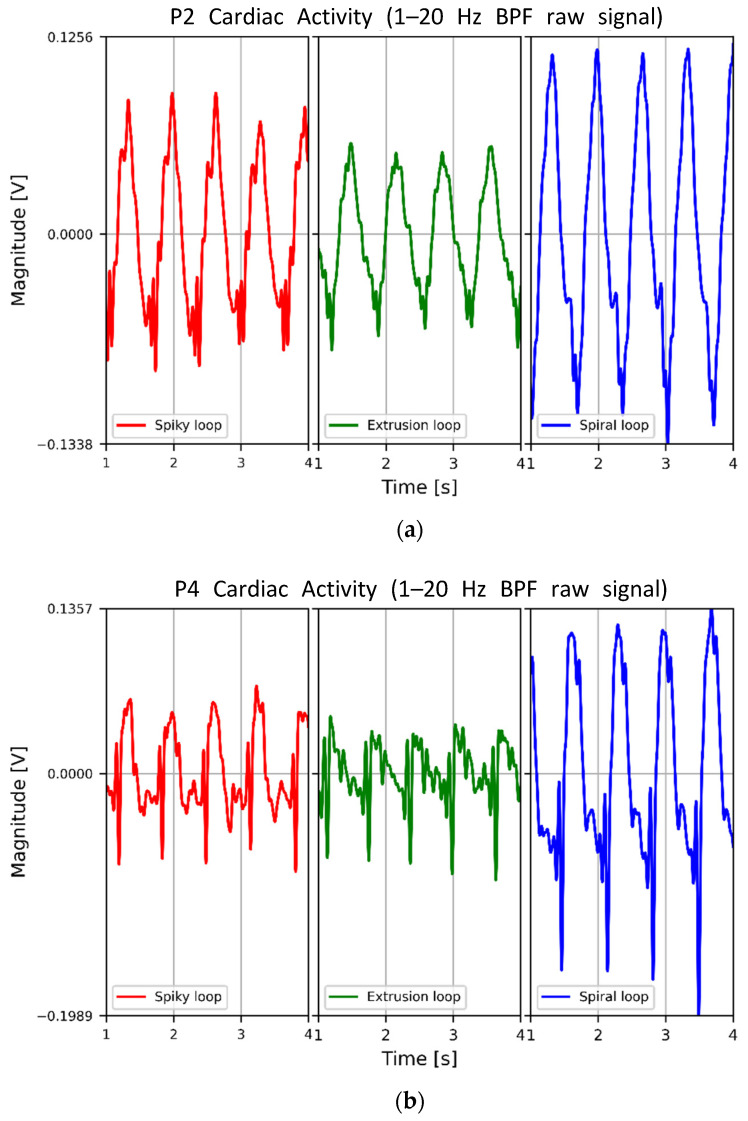

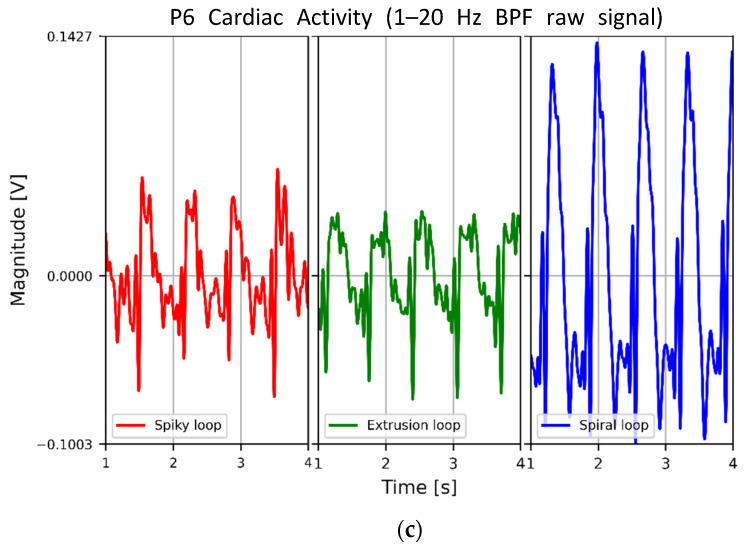
Differences in the acquired signals according to the shape of the fabric loop sensor for each measurement position: (**a**) P2, (**b**) P4, and (**c**) P6 (spiral > spiky > extrusion).

The waveforms of the signal filtered by a bandpass filter (BPF, 1–20 Hz) from the raw signal measured using the fabric loop sensor and the ECG signal acquired from the Ag/AgCI electrode were qualitatively analyzed. Figure 6 compares the raw signals detected at each measurement position, P2, P4, and P6, at different times on the same scale. The signals measured using spiral loop sensors at all three measurement positions showed the largest changes in magnitude compared to the other loop sensors. Conversely, the signals measured using the spiky and extrusion loop sensors exhibited similar changes in magnitude.

**Figure 7:**
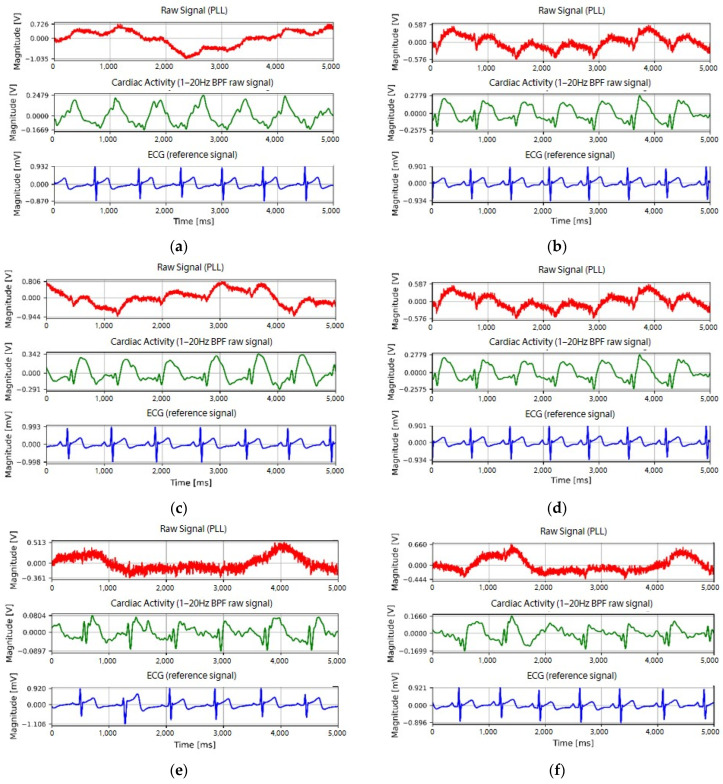
Cardiac activity signals according to the shape and measurement position of the fabric loop sensor: spiral loop sensor at (**a**) P2, (**b**) P4, and (**c**) P6; (**d**) spiral, (**e**) extrusion, and (**f**) spiky loop sensors at P4.

Examples of the waveforms of the cardiac activity signals measured on subject A are presented in Figure 7. The upper waveform of the graph is the raw signal detected using the developed fabric loop sensor and the waveform in the middle is the signal filtered by a bandpass filter. The bottom waveform shows the reference signal detected by the clinical Ag/AgCl electrode. Figure 7a–c show the signals when the spiral loop fabric sensor was used to acquire measurements at positions P2, P4, and P6, respectively; Figure 7d–f show the signals when the measurements were acquired at the P4 position using the spiral, extrusion, and spiky loop sensors, respectively. Regarding the shape of the signals, the reference signal detected by the Ag/AgCl electrode showed the largest peak at the R-peak point. The shape of the signal detected by the fabric loop sensor showed a change similar to the QRS pattern of the ECG at the R-peak point of the reference signal. However, compared to the change in the overall signal, the magnitude was initially small, followed by a large change. Regarding the signal difference according to the measurement positions (P2, P4, P6) of the fabric loop sensor, a change in the signal at all three positions was observed. However, the signal measured at P2 showed a smaller QRS pattern than those measured at P4 and P6. The signal difference according to the shape of the fabric loop sensor showed regular changes regardless of the shape; however, the width of the change was larger in the spiral loop sensor than in the spiky or extrusion loop sensors. Unlike that of the spiral loop sensor, the signals detected by the other two types of sensors were mixed with a small high-frequency signal. Therefore, by qualitatively analyzing the cardiac activity signal, the P4 and P6 positions were more effective than P2 in terms of fabric electrode placement. The spiral shape of the fabric loop sensor was evaluated as being more effective than the spiky or extrusion loop shapes.

**Figure 8:**
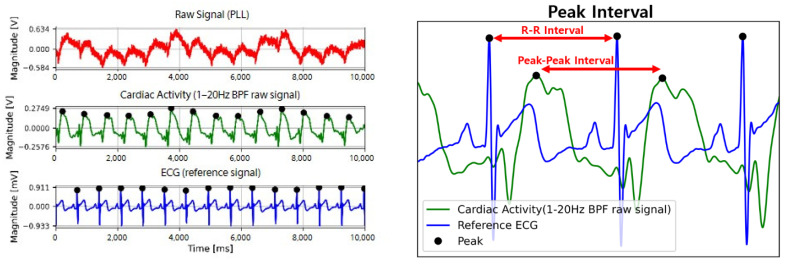
Cardiac activity signals measured using clinical ECG signals and spiral loop sensor at P4 (R-peak–PLL-peak analysis).

Figure 8 shows the intervals according to the order of each R-R peak for a more quantitative comparative analysis. The peak points of the ECG and filtered PLL signals were identified, and the time of appearance and magnitudes of the peaks were compared. The peak point of the filtered PLL signal was clearly shown, and the peak points of the ECG and filtered PLL signals were slightly different. The PLL signal peak appeared a little later than that of the ECG signal (Figure 8). Overall, the signal detected from the fabric loop sensor peaked after a slight delay compared to the ECG signal; however, the interval between the peaks was synchronized.

**Figure 9:**
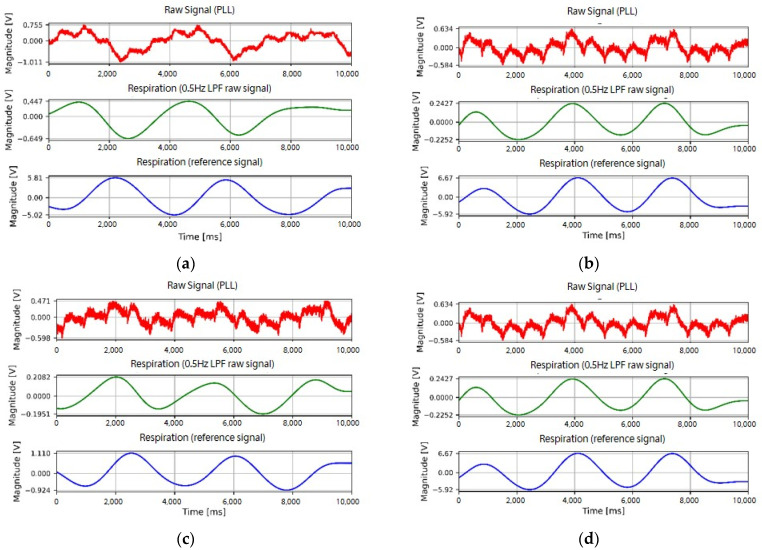

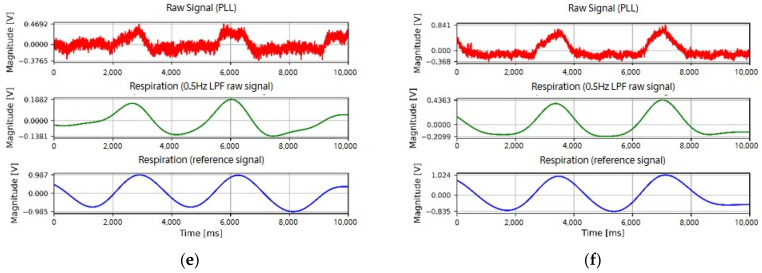
Respiration signals according to the shape and measurement position of the fabric loop sensor: spiral loop sensor at (**a**) P2, (**b**) P4, and (**c**) P6; (**d**) spiral, (**e**) extrusion, and (**f**) spiky loop sensors at P4.

An example of the respiratory signal waveform measured from subject A is presented in Figure 9. The upper waveform is the raw signal detected by the developed fabric loop sensor, and the middle waveform is the signal filtered by the bandpass filter. The bottom waveform is the reference signal measured in the abdomen using BIOPAC. Figure 9a–c are the signals measured at P2, P4, and P6 using a spiral loop sensor, respectively, whereas Figure 9d–f are the signals measured at position P4 using the spiral, extrusion, and spiky loop sensors, respectively. As shown in Figure 9, respiratory signals could be detected for all sensor shapes and measurement positions. However, the respiratory reference signal measured by BIOPAC was from the abdomen, whereas that of the fabric loop sensor measured was from the chest, thus resulting in a phase difference. In particular, the signal measured at P2 showed a large phase difference compared to the other positions regardless of the shape of the fabric loop sensor.

**Figure 10:**
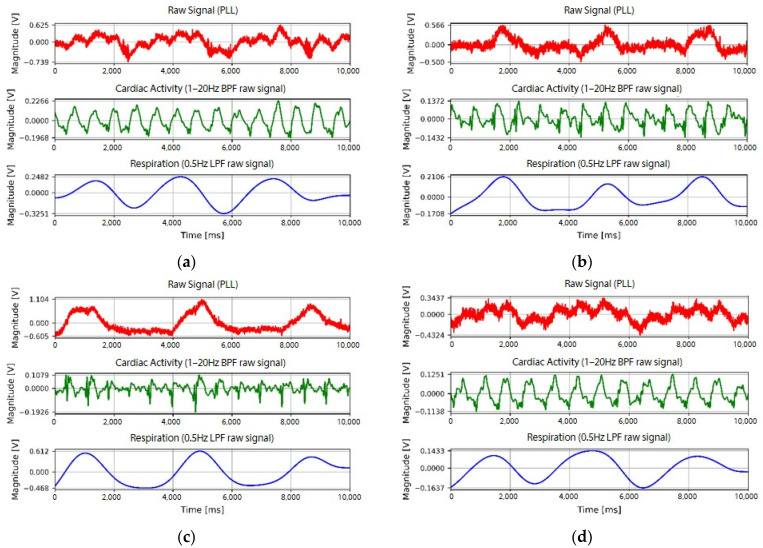

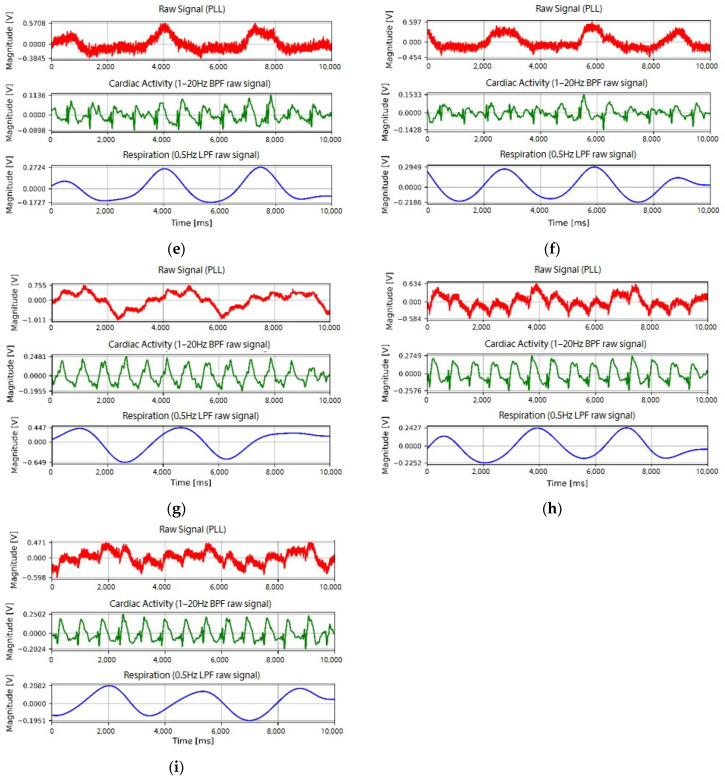
Cardiac activity and respiratory signals detected in subject A: spiky loop sensor at (**a**) P2, (**b**) P4, and (**c**) P6; extrusion loop sensor at (**d**) P2, (**e**) P4, and (**f**) P6; and spiral loop sensor at (**g**) P2, (**h**) P4, and (**i**) P6.

As a result, it was possible to detect cardiac activity and respiration signals at all measurement positions (P2, P4, and P6) using the different fabric loop sensors (spiky, extrusion, and spiral) despite the slight difference in performance, as shown in Figure 10.

### 5.2. Evaluation of Sensing Performance through Quantitative Statistical Analysis

Quantitative analysis was performed to verify whether there was a difference in sensing performance depending on the shape characteristics and measurement position of the fabric loop sensor. The cardiac activity signals acquired using the fabric loop sensor and clinical Ag/AgCl electrodes were analyzed using Anaconda Navigator 2.0.3 (Spyder 4.2.5) and the SNR value was evaluated. The Kruskal–Wallis test, a nonparametric difference test method, was conducted using the SNR value as a parameter to determine whether there was a performance difference among the nine combinations of the shape and measurement position of the fabric loop sensor (Table 2). The analysis revealed a statistically significant difference among the nine cases (*p* = 0.000 (<0.05)). A Bonferroni post hoc test was performed to determine whether there was a difference among the nine combinations due to the shape and measurement position of the fabric loop sensor. Significant differences were observed for the measurement positions P4 and P6 when using a spiral loop sensor (*p*-value = 0.000 (<0.05)), that is, the cardiac activity signal could be measured most effectively at the P4 and P6 positions using the spiral loop sensor. There were no differences in the performances of the other combinations. In addition, Table 3 lists the results of the Kruskal–Wallis test to analyze the difference in the measurement performance according to the shape features of the fabric loop sensor. A significant difference in performance was found according to the three shapes (spiky, extrusion, and spiral) of the fabric loop sensor (*p*-value = 0.000 (<0.05)). A Bonferroni post hoc test was also performed to identify the specific sensors for which the differences were observed. The spiral loop sensor had higher sensing efficiency than the spiky or extrusion loop sensors. No significant differences were found between the spiky and extrusion loop sensors. Table 4 lists the results of the Kruskal–Wallis test to analyze the difference in the sensing performance of cardiac activity according to the measurement location. A significant difference in performance was observed corresponding to the three measurement positions P2, P4, and P6 (*p*-value = 0.000 (<0.05)). A Bonferroni post hoc test was performed to verify whether there was a difference in performance due to the three measurement positions. The performance was better when the cardiac activity was measured at the P4 or P6 position than at the P2 position. No significant differences were observed between the P4 and P6 positions. Finally, it was verified that the highest quality of the measurements of cardiac activity and respiration signals could be simultaneously acquired using the spiral loop sensor at P4 or P6.

## 6. Conclusions

In this study, a noncontact fabric loop sensor based on magnetic-field-induced conductivity, which can simultaneously detect cardiac activity and respiration signals, was developed. In addition, we attempted to identify the optimal shape and measurement location of the fabric loop sensor to simultaneously detect high-quality cardiac activity and respiration signals. First, we qualitatively compared and analyzed the waveform of the ECG signal obtained from the clinical Ag/AgCI electrode with the signal acquired by filtering the raw signal measured by the fabric loop sensor with a BPF (1–20 Hz). Although there was a slight difference in sensing performance, the three types of fabric sensors were able to effectively detect cardiac activity and respiration signals at the measurement positions.

Next, a quantitative analysis was performed to verify whether there was a difference in sensing performance depending on the shape characteristics and measurement position of the fabric loop sensor. A nonparametric analysis was conducted to determine whether there was a difference in sensing performance among the nine combinations of shape and measurement location of the fabric loop sensor in terms of the SNR value. It was statistically verified that the highest measurement quality for the cardiac activity and respiration signals was obtained using the spiral loop sensor at P4 or P6. In conclusion, the developed sensors can simultaneously detect heart activity and respiratory signals at all three measurement positions. In the future, additional research will be needed to address motion artifacts, which commonly appear in biosignal measurements using a noncontact method. When the electrode position deviates from its original position owing to various factors during biosignal measurement, noise is introduced on the measured signal, and its accuracy becomes degraded. Therefore, the removal and reduction of motion artifacts is an important subject that must be considered in wearable products that measure biosignals daily in real time. Attempts to solve this noise have been implemented in terms of circuit and algorithm development. However, in future studies, solutions using clothing structure design are needed. To this end, we intend to develop diverse types of upgraded fabric sensors and improve the selection of variables for analysis. In addition, based on the results of these follow-up studies, an apparel-based wearable platform system with integrated fabric sensors will be developed and methods to improve its performance will be analyzed. Ultimately, we intend to develop smart clothing technology that can acquire high-quality heart-activity signals without time and space constraints through research on the convergence of smart clothing and biosignal measurement. This can be utilized as an important indicator of the user’s physical condition for the proactive prevention and treatment of diseases.

## Figures and Tables

**Table 1:** Shapes and specifications of the three types of fabric loop sensors.

	Shape	Spiky Loop	Extrusion Loop	Spiral Loop
**Specification**		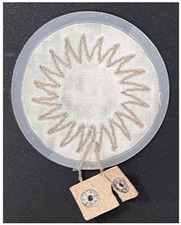	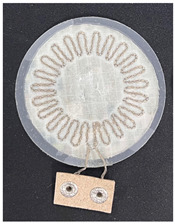	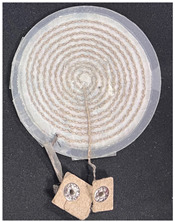
Diameter		50 mm	50 mm	50 mm
Inductance (H @ 2 MHz)		533 nH	588 nH	1.90 μH

**Table 2:** Efficiency evaluation of cardiac activity signals according to the sensor shape and measurement position.

Sensor Shape and Measurement Position	N	Mean (SD)	Kruskal–Wallis	*p*-Value	Bonferroni
Spiral loop @ P2	60	5.0560 (2.3610)	53.282	0.000 *	Spiral loop @ P4 > Spiral loop @ P2, Extrusion loop @ P2, Extrusion loop @ P4, Extrusion loop @ P6, Spiky loop @ P2, Spiky loop @ P4, Spiky loop @ P6 Spiral loop @ P6 > Extrusion loop @ P2, Extrusion loop @ P4, Extrusion loop @ P6, Spiky loop @ P2, Spiky loop @ P4, Spiky loop @ P6 Spiral loop @ P4, Spiral loop @ P6 > Spiral loop @ P2, Extrusion loop @ P2, Extrusion loop @ P4, Extrusion loop @ P6, Spiky loop @ P2, Spiky loop @ P4, Spiky loop @ P6
Spiral loop @ P4	60	7.1478 (2.6055)			
Spiral loop @ P6	60	7.0922 (2.6823)			
Extrusion loop @ P2	60	4.2866 (2.0868)			
Extrusion loop @ P4	60	5.3880 (2.9638)			
Extrusion loop @ P6	60	5.2741 (2.2762)			
Spiky loop @ P2	60	5.0204 (2.4501)			
Spiky loop @ P4	60	5.2977 (2.8500)			
Spiky loop @ P6	60	5.3863 (2.7156)			

* *p*-value < 0.05.

**Table 3:** Efficiency evaluation of cardiac activity signals according to the shape of the fabric loop sensor.

Sensor Shape	N	Mean (SD)	Kruskal–Wallis	*p*-Value	Bonferroni
Spiral loop sensor	180	6.4320 (2.7201)	27.307	0.000 *	Spiral loop sensor > Extrusion loop sensor Spiral loop sensor > Spiky loop sensor
Extrusion loop sensor	180	4.9829 (2.5068)			
Spiky loop sensor	180	5.2348 (2.7050)			

* *p*-value < 0.05.

**Table 4:** Efficiency evaluation of cardiac activity signals according to the measurement position.

Measurement Position	N	Mean (SD)	Kruskal–Wallis	*p*-Value	Bonferroni
P2	180	4.7877 (2.3190)	18.291	0.000 *	P4 > P2 P6 > P2
P4	180	5.9445 (2.9222)			
P6	180	5.9175 (2.7225)			

* *p*-value < 0.05.
